# Translation and transcultural validation of the Dutch hospital for special surgery paediatric functional activity brief scale (HSS Pedi-FABS)

**DOI:** 10.1186/s12891-021-04729-0

**Published:** 2021-10-06

**Authors:** Martijn Dietvorst, Tessa M. van de Kerkhof, Rob P. A. Janssen, Linda E. van den Berg, M. C. ( Marieke) van der Steen

**Affiliations:** 1 Department of Orthopaedic Surgery & Trauma, Máxima Medical Centre, Eindhoven, the Netherlands; 2 Faculty of medical sciences, Radboud Institute for Health Sciences, Radboud University, Nijmegen, the Netherlands; 3 Orthopaedic Biomechanics, Department of Biomedical Engineering, Eindhoven University of Technology, Eindhoven, the Netherlands; 4 Chair Value-Based Health Care, Department of Paramedical Sciences, Fontys University of Applied Sciences, Eindhoven, the Netherlands; 5 Department of Orthopaedics & Sports Medicine, Erasmus University Medical Centre, Rotterdam, the Netherlands; 6 Department of Orthopaedic Surgery & Trauma, Catharina Hospital Eindhoven, Eindhoven, the Netherlands

**Keywords:** HSS Pedi-FABS, Paediatric, Adolescent, Activity scale, Validation study

## Abstract

**Background:**

There is a need for a validated simple Dutch paediatric activity scale. The purpose was to translate and transculturally validate the Dutch Hospital for Special Surgery Paediatric Functional Activity Brief Scale (HSS Pedi-FABS) questionnaire in healthy children and adolescents.

**Methods:**

The original HSS Pedi-FABS was translated forward and backward and was transculturally adapted after performing a pilot study among children and professionals. The final version of the Dutch HSS Pedi-FABS was validated in healthy children and adolescents aged 10 to 18 years old. Children who had any condition or injury limiting their normal physical activity were excluded. The interval between the first questionnaire T0 (HSS Pedi-FABS, Physical Activity Questionnaire for children or adolescents (PAQ-C/A) and Tegner activity scale) and the second questionnaire T1 (HSS Pedi-FABS) was 2 weeks. Construct validity, interpretability and reliability were evaluated. Content validity was evaluated through cognitive interviews among a smaller group of children and through a questionnaire among professionals.

**Results:**

To evaluate content validity, 9 children and adolescents were interviewed, and 30 professionals were consulted. Content validity among professionals showed a relevance of less than 85% for most items on construct. However, content validity among children was good with a 92% score for item relevance. Readability was scored at a reading level of 11- to 12-year-olds. The validation group consisted of 110 healthy children and adolescents (mean age of 13.9 years ±2.6). Construct validity was considered good as 8 out of 10 hypotheses were confirmed. The Dutch HSS Pedi-FABS showed no floor or ceiling effect. Analysis of the internal consistency in the validation group resulted in a Cronbach’s alpha of 0.82. Test-retest reliability was evaluated among 69 children and adolescents and revealed an Intraclass Correlation Coefficient (ICC) of 0.76.

**Conclusion:**

The Dutch HSS Pedi-FABS showed good psychometric properties in a healthy Dutch paediatric and adolescent population. Limitations of the current Dutch HSS Pedi-FABS are content validity on construct of items reported by professionals.

**Supplementary Information:**

The online version contains supplementary material available at 10.1186/s12891-021-04729-0.

## Background

Physical activity provides important health benefits for children and adolescents. Unfortunately, injuries related to physical activity are common, especially in single sports specialization [1–3]. With 42,000 sports and physical activity-related injuries seen among 5–14 year-old children in Dutch hospitals every year, sports injuries are a substantial public health issue [1, 4].

The level of physical activity is increasingly recognized as both an important prognostic factor and outcome variable in orthopaedics [5]. A simple validated outcome measure is important to determine physical activity in children and adolescents. Physical activity can be assessed with both objective and subjective measures. Objective measures such as accelerometers and heart-rate monitors provide highly reproducible and accurate data on physical activity but are often rather expensive, time-consuming, and may require technical expertise [6]. Self-reported measures, such as questionnaires, are often used to assess physical activity in children and adolescents because of their advantages, such as low costs, minimal participant burden, and easy administration. However, problems may arise with the length of the questionnaire, understanding the questions, or accurately recalling physical activity especially in a young target population [7].

Multiple self-reported activity scales already exist in the orthopaedic field [8, 9]. However, current scales are often aimed at children with a specific disability [8]. The existing activity scales for children are long, time consuming and specific to activity, sport and/or joint [7, 8, 10]. Long questionnaires may lead to questionnaire fatigue [8, 11]. Global use of activity-specific scales may also be limited due to cultural biases [8, 12, 13]. Moreover, a recent review by Hidding et al. [14] argued that there is a lack of physical activity questionnaires with excellent validity and reliability [14]. To date, the Physical Activity Questionnaire – Children or Adolescent (PAQ-C and PAQ-A) are the only validated Dutch questionnaires to assess physical activity in children and adolescents [14, 15]. These questionnaires have 9 to 10 items including a checklist of 23 sports and are therefore long and time-consuming. In 2013, the Hospital for Special Surgery Paediatric Functional Activity Brief Scale (HSS Pedi-FABS) was developed to assess the physical activity level in children and adolescents aged 10 to 18 years old [8]. The HSS Pedi-FABS is a simple, validated paediatric activity scale, which may be useful to evaluate physical activity level as a prognostic factor in clinical outcome research [16]. It has excellent scale reliability, robust construct validity, and shows no floor or ceiling effects [8]. Besides, the HSS Pedi-FABS has recently been recommended by the 2018 International Olympic Committee (IOC) consensus statement and will be used as activity-rating scale in the Paediatric Anterior Cruciate Ligament Monitoring Initiative (PAMI) [17]. This European initiative launched by the European Society for Sports Traumatology, Knee Surgery and Arthroscopy (ESSKA) aims to create a pan-European system to collect and analyse data to provide stronger scientific evidence in paediatric ACL injury treatment [17–19]. Yet, the HSS Pedi-FABS is currently available in English and Italian [8, 18]. Therefore, it is crucial to transculturally validate the Dutch HSS Pedi-FABS. It is hypothesized that the Dutch HSS Pedi-FABS has adequate psychometric properties in a healthy paediatric and adolescent population, comparable to the psychometric properties of the original HSS Pedi-FABS [8].

## Methods

### Translation procedure

Translation of the original HSS Pedi-FABS, which is published by Fabricant et al. [8], was performed using a forward-backward translation procedure [20]. The HSS Pedi-FABS was translated from English into Dutch by two native Dutch speakers. Translations were compared, discrepancies between them were discussed and a preliminary version of the Dutch HSS Pedi-FABS was established. Subsequently, this preliminary version was translated back into English by two independent English native speakers who were unfamiliar with the original questionnaire. The translated version was compared to the original version of the HSS Pedi-FABS to check for similar item content. Differences and inconsistencies were discussed and adjustments were made to form the pre-final version. This pre-final version was evaluated as a pilot among children and professionals for cross-cultural adaptation which resulted in minor adjustments. The developer of the original HSS Pedi-FABS was consulted to discuss cross-cultural adaptations [8]. Finally, the Department of Patient Communication at the Máxima Medical Centre evaluated this pre-final version. Some linguistic adjustments were made, and the final version of the Dutch HSS Pedi-FABS was established.

### Participants

The study population consisted of the content validity group and the validation group. The content validity group consisted of two subgroups: target population and professionals. Participants of the content validity target population were recruited at a sports club and through a personal network. Physically active children aged 10 to 18 years old were included in this group. Professionals from relevant disciplines were recruited from four Dutch teaching hospitals: Máxima Medical Centre Eindhoven/Veldhoven, VieCuri Hospital Venlo, Maastricht University Medical Centre and Erasmus University Medical Centre Rotterdam. The validation group was recruited through primary and secondary schools in the Netherlands and at the out-patient department of the paediatric orthopaedic clinic at the Máxima Medical Centre and Erasmus University Medical Centre. Children or adolescents aged 10 to 18 years were included in this group. Children who had any condition or injury limiting their normal physical activity were excluded.

### Study procedure

**Fig. 1:**
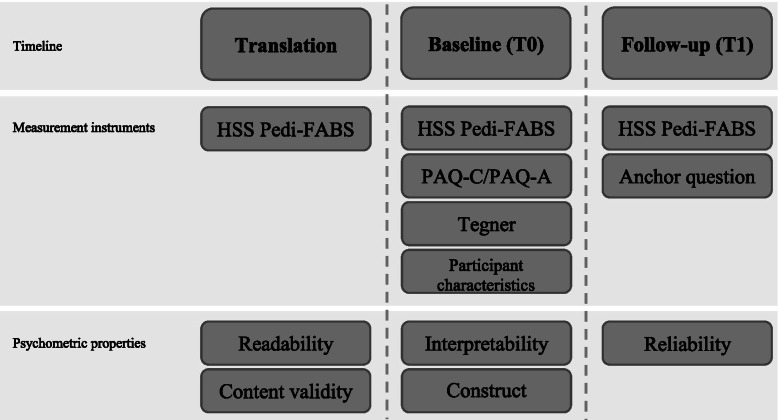
Study procedure. “HSS Pedi-FABS = Hospital for Special Surgery Paediatric Functional Activity Brief Scale”; “PAQ-C/A = Physical Activity Questionnaire – Children or Adolescents”. Overview of study design

Figure 1 shows the study procedure of the translation and validation of the Dutch HSS Pedi-FABS. After translation, content validity was assessed through cognitive interviews in participants representing the target population and through questionnaires in professionals [21]. Interpretability and construct validity were evaluated within the validation group. Participants received an information letter together with a set of questionnaires at school (T0). Assistance in completing the questionnaire by the parents was allowed for any reason. If the participants completed the baseline questionnaires, they were asked to fill out the Dutch HSS Pedi-FABS again by email or post 2 weeks later (T1) and answer the anchor question “Did your level of physical activity change since you completed the previous questionnaires (± 2 weeks ago)?”. Responses of participants reporting stable level of activity were used to assess reliability of the Dutch HSS Pedi-FABS.

### Questionnaires

At baseline (T0), children and adolescents within the validation group completed the HSS Pedi-FABS, PAQ-C or PAQ-A, Tegner activity scale, and questions concerning age, school, self-reported weekly participation in sports and self-reported level of competition.

The HSS Pedi-FABS questionnaire is an 8-item metric to assess physical activity in children and adolescents between 10 and 18 years old [8]. The questionnaire consists of 6 Likert-based items regarding frequency of activities, one item on the level of sports and one item on supervision [8, 18]. Scores range from 0 to 30, with a higher score indicating more physical activity [8, 18].

The PAQ-C and PAQ-A are the only validated Dutch questionnaires to assess physical activity in children and adolescents [14, 15]. The PAQ-C was originally designed for children aged 8 to 14 years and consists of 10 questions [15]. The PAQ-A was designed for adolescents aged 14 to 20 years and consists of 9 questions [15]. Both PAQ questionnaires contain one question in the form of a checklist of common sports and activities which are scored on frequency of participation [15]. Subsequently, the mean is calculated which results in the total score ranging from 1 (low activity) to 5 (high activity) [15].

Although the Tegner activity scale is not validated in the paediatric population, it is often used as an activity scale for children and adolescents [22]. The Tegner activity scale is a 1-item activity scale ranging from 0 (no activity) to 10 (high activity) and is widely used as an activity rating system for a variety of knee disorders [13, 23, 24].

### Readability

Readability of the Dutch HSS Pedi-FABS was assessed with the Dutch version of the Flesch reading ease (FRE) test [25]. A score of 0 reflects academic language while a score of 100 reflects the reading level of children in 4th grade (age 9–10 years). The aim was to attain a readability score between 60 and 80 points, which reflects the reading level of children aged 11 to 13 years old.

### Content validity

#### Target population

Relevance, comprehensiveness and comprehensibility were evaluated through cognitive interviews with children [26]. All interviews were conducted by one researcher (TK) and were audio-recorded for transcription. A semi-structured interview guide was used containing predefined probe questions which addressed comprehension of the instructions, items, recall period, and response options [27]. All items of the HSS Pedi-FABS were also rated on relevance, and participants were asked to suggest potential missing concepts. Parents were not asked for input on the scale. Interviews were transcribed verbatim and analysed by one researcher (TK) using ATLAS.ti version 8.4 (Scientific Software Development GmbH, Berlin, Germany). Cognitive codes were applied using the Problem Classification Coding Scheme (CCS), which consists of five categories: comprehension and communication; memory retrieval; judgement and evaluation; response selection; and other (see Supplementary file 1) [28]. A sixth category containing four codes was added to determine relevance.

#### Professionals

Professionals from relevant disciplines (orthopaedic surgeons, residents in orthopaedic surgery, physiotherapists, sports physicians, rehabilitation physicians, and trauma surgeons) rated the relevance and comprehensiveness of the Dutch HSS Pedi-FABS. A questionnaire was used to evaluate the relevance of each item for both the target population and the construct; the response options and recall period were rated on appropriateness. Comprehensiveness was addressed by asking whether important concepts or items were missing in the questionnaire.

Items were considered relevant for the target population or construct if at least 85% of professionals rated them as relevant. Content validity of the Dutch HSS-Pedi FABS was rated as sufficient if at least 85% of the items were considered relevant by both professionals and participants [21].

### Interpretability

Interpretability was assessed by examining the distribution of HSS Pedi-FABS scores at T0, including the mean and standard deviation (SD). Moreover, floor and ceiling effects were evaluated and considered present if more than 15% of the participants scored either the lowest or highest score possible [8, 21, 29]. A positive rating of interpretability of the HSS Pedi-FABS was given if floor and ceiling effects were absent.

### Construct validity

Hypothesis testing was used to assess construct validity; criterion validity was not evaluated since no gold standard is available for questionnaires on physical activity. Hypotheses were defined about the relationship between the HSS Pedi-FABS and outcome measures which measure either the same or a different construct (convergent or discriminative validity, respectively). These hypotheses were formulated by a panel consisting of experts and based on the study of Fabricant et al. [8]

To evaluate convergent validity, a correlation of r ≥ 0.50 was expected between the HSS Pedi-FABS scores and a) the PAQ scores (total), b) PAQ-C scores, c) PAQ-A scores, d) the Tegner activity score, e) self-reported hours of weekly participation in sports, and f) the level of competition, all assessed at baseline (T0). Age and BMI were expected not to correlate (< 0.30) with scores of the HSS Pedi-FABS which reflects divergent validity. Correlations of the hypotheses confirming convergent validity should be at least 0.1 higher than the correlations that indicate discriminative validity [26]. The latter was operationalized as two hypotheses: hypotheses conforming convergent validity should be at least 0.1 higher than age (hypothesis 9) and BMI (hypothesis 10). Construct validity of the HSS Pedi-FABS was considered good if at least 75% of the predefined hypotheses were confirmed [21].

### Reliability

Internal consistency, test-retest reliability and measurement error were evaluated as measurement properties of reliability [21]. All participants within the validation group were included for analysis of internal consistency. All participants who completed the baseline questionnaires were invited to complete the Dutch HSS Pedi-FABS a second time 2 weeks (T1) after completion of T0. Only participants who reported no change in their activity pattern during the interval period were included in the test-retest analysis.

### Comparison between HSS Pedi-FABS versions

The psychometric properties of the original, the Italian and Dutch HSS Pedi-FABS were compared.

### Statistical analysis

Statistical analyses were performed with IBM SPSS Statistics 24. Descriptive statistics were used to describe baseline characteristics of the participants. The aim was to include at least 7 participants and 30 professionals to evaluate relevance and to ensure excellent content validity [21, 26]. To assess construct validity, interpretability, and reliability, at least 100 participants needed to be included in the validation group [26]. Shapiro-Wilk test was used as test for normality of the baseline characteristics and HSS Pedi-FABS outcomes in the validation group [30]. Spearman rank correlations were calculated to assess construct validity. To determine internal consistency, Cronbach’s alpha was calculated. Test-retest reliability was evaluated by means of a two-way random effects model of Intraclass Correlation Coefficient (ICC) in absolute agreement. Cronbach’s alpha and ICC coefficients of 0.70 or higher are considered to reflect good reliability [20]. The Standard Error of Measurement (SEM) was calculated as SEM = SD * √(1 – reliability), where the ICC reflects reliability [21, 31]. The Smallest Detectable Change (SDC) was defined as 1.96 * √2 * SEM [32]. The significance level was set at 5% for all statistical analyses.

### Ethical approval

This validation study was approved by the local Medical Ethics Committee (METC) of the Máxima Medical Centre [N18.168] and Erasmus University Medical Centre [MEC-2020-0278]. The developer of the original HSS Pedi-FABS was informed and gave permission for publication of the Dutch HSS Pedi-FABS. All participants gave written informed consent and their parents or legal guardians if necessary (in case of age < 16 years).

## Results

The content validity population consisted of 9 participants. A total of 132 children and adolescents were included for the validation study, of which 22 participants reported a condition or injury limiting their normal physical activity and were excluded for analysis. In Table 1, the characteristics of the content validity population and validation population are shown. 39.1% of the children received assistance from parents in completing the questionnaires, of which 73% was aged 10 to 12 years. The reasons or types of assistance by the parents were not evaluated.

**Table 1:** Baseline characteristics of the content validity population and the validation population at T0 and T1

	Content validity	Validation	
	Participants (***n*** = 9)	T0 Participants (***n*** = 110)	T1 Participants (***n*** = 69)
Age, mean ± SD, y	13.4 ± 2.4	13.9 ± 2.6	13.7 ± 2.5
Sex, No. (%)			
Female	7 (78)	60 (55)	38 (51)
Male	2 (22)	50 (46)	37 (49)
BMI, mean ± SD, kg/m^2^	19.4 ± 2.3	18.3 ± 2.7	18.1 ± 2.8
Weekly participation in sports at a sports club, mean ± SD, h		4.3 ± 3.1	4.0 ± 2.9
Weekly participation in sports without sports club, mean ± SD, h		4.3 ± 4.3	4.0 ± 2.3
Days per week with at least 1 hour of physical activity, No. (%)			
Almost never		1 (1)	0 (0)
1 day per week		3 (3)	3 (4)
2 days per week		3 (3)	2 (3)
3 days per week		15 (14)	9 (12)
4 days per week		11 (10)	5 (7)
5 days per week		22 (20)	15 (20)
6 days per week		24 (22)	18 (24)
Every day		30 (27)	22 (30)
Missing		1 (1)	1 (1)
Competition level, No. (%)			
Recreational		34 (31)	23 (32)
Regional		64 (58)	45 (63)
National		5 (5)	3 (4)
International/elite		3 (3)	1 (1)
Missing		4 (4)	3 (4)

*BMI* Body mass index, *h* Hours, *No.* Number, *SD* Standard deviation, *y* Years

### Readability

The readability level was estimated at 71 which corresponds to a readability level of 11- to 12-year-old children.

### Content validity

#### Target population

The interviews (*n* = 9) yielded 32 different codes, 28 as defined in the CCS and 4 extra codes to evaluate the relevance of each item (Supplementary file 1). In total, 127 times a code was applied; 54 times this was a relevance code and 73 times a code from the CCS (Table 2). Considering the relevance of the items, 92.3% (50/54) was considered relevant by the participants of which 69% (37/54) was evaluated as highly relevant; in three cases (5.6%) it was unclear whether the item was considered relevant and only once an item was indicated as not relevant (1.9%). Over half of the applied codes from the CCS (45/73) were classified in the comprehension and communication category (Table 2). The items that were most often regarded as complex or vague were cutting and pivoting. Sometimes, participants struggled with the difference between endurance and duration. Furthermore, a few participants had difficulty estimating the frequency of the requested item. It should however be noted that some codes were applied multiple times on the same item in the same interview, due to persistent problems in comprehensibility and questions for further explanation in some of the interviews. This added to the high frequency of applied codes in the comprehension and communication category. The problem codes for each category of comprehension and communication are shown in Supplementary file 1.

**Table 2:** Problem codes found in the interviews

Problem codes	Frequency
Classification Coding Scheme	73
1. Comprehension and Communication	45
2. Memory Retrieval	8
3. Judgment and Evaluation	10
4. Response Selection	6
5. Other	4
Relevance	54
1. Highly relevant	37
2. Somewhat relevant	13
3. Not relevant	1
4. Unclear	3
**Total**	127

#### Professionals

**Fig. 2:**
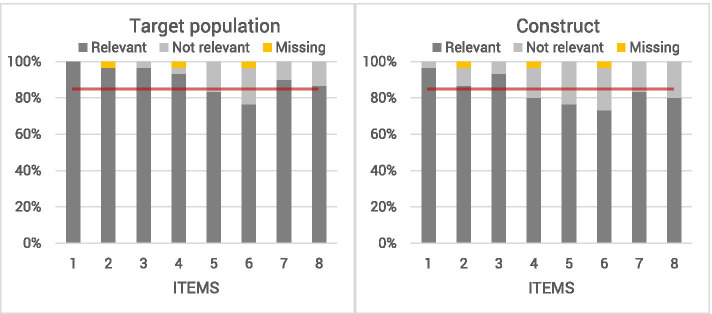
Relevance for the target population (left) and the construct (right) rated by professionals (*n* = 30). Graph of relevance for target population and construct by professionals

The 30 professionals consisted of 9 orthopaedic surgeons, 2 trauma surgeons, 1 sports physician, 13 residents and 5 physiotherapists. Figure 2 shows the relevance according to the professionals for each item with regard to the target population and construct. Item 5 and 6 were considered relevant by less than 85% of the professionals for both the target population and construct. Item 4, 7 and 8 also achieved a relevance score of less than 85% for the construct. The recall period of 1 month was considered “good” by 67% of the professionals, 20% found that the recall period was too long and 13% that it was too short. Almost half of all professionals suggested that additional items were necessary to measure physical activity. The most frequently suggested additional items were cycling to school (*n* = 4), other physical activities such as playing outside or physical education (n = 4) and injuries (*n* = 3).

Overall, content validity among the target population was considered good and among professionals acceptable for relevance of the target population but insufficient for relevance of the construct.

### Interpretability

Mean scores of the questionnaires assessed at baseline, together with floor and ceiling effects are presented in Table 3. HSS Pedi-FABS scores (mean score: 17.6 ± 6.2) were not normally distributed. The HSS Pedi-FABS, PAQ-C, PAQ-A and Tegner activity scale showed no floor or ceiling effect. The interpretability of the HSS Pedi-FABS was considered as good.

**Table 3:** Scores for each scale, floor and ceiling effects

	Mean score ± SD	Range	Lowest score	Highest score
HSS Pedi-FABS (n = 110)	17.6 ± 6.3	0–29	1%	0%
PAQ-C (*n* = 42)	3.0 ± 0.5	1.8–4.3	0%	0%
PAQ-A (*n* = 67)	2.6 ± 0.5	1.5–3.5	0%	0%
Tegner (*n* = 108)	6.5 ± 2.3	1–10	3%	2%

*HSS Pedi-FABS* Hospital for Special Surgery Paediatric Functional Activity Brief scale, *PAQ-A/C* Physical Activity Questionnaire – Adolescents/Children, *SD* standard deviation

### Construct validity

Spearman rank correlations were calculated to evaluate the predefined hypotheses for the construct validity, as shown in Table 4. Except for the PAQ-C and weekly participation in sports, the hypotheses regarding convergent validity were confirmed. Both hypotheses on discriminative validity were also confirmed. Furthermore, all correlations evaluating convergent validity were at least 0.1 higher than the correlation of age or BMI with the HSS Pedi-FABS. Eight out of the ten (80%) hypotheses were confirmed, indicating a good construct validity.

**Table 4:** Spearman rank correlations for construct validity

	HSS Pedi-FABS	Hypothesis confirmed?
**Convergent validity (r ≥ 0.50)**		
PAQ (*n* = 109)	**0.500**	Yes
PAQ-C (n = 42)	0.105	No
PAQ-A (n = 67)	**0.588**	Yes
Tegner (n = 108)	**0.666**	Yes
Weekly participation in sports	0.409	No
Competition level	**0.649**	Yes
**Discriminative validity (r < 0.30)**		
Age	**−0.017**	Yes
BMI	**−0.233**	Yes
**Differences in correlations between convergent and discriminative validity (*r*** **> 0.1)**		
PAQ-C/A; Tegner; Weekly participation; Competition level versus Age	**All > 0.1**	Yes
PAQ-C/A; Tegner; Weekly participation; Competition level versus BMI	**All > 0.1**	Yes

*BMI* Body mass index, *HSS Pedi-FABS* Hospital for Special Surgery Paediatric Functional Activity Scale, *PAQ-A/C* Physical Activity Questionnaire – Adolescent/Children

### Reliability

Analysis of the internal consistency of the HSS Pedi-FABS in 110 children, resulted in a Cronbach’s alpha of 0.82, indicating a good internal consistency.

Of the 110 children that were included at baseline, 89 children responded at follow-up. For analysis, 14 children were excluded because of altered activity patterns and six children due to technical errors at T1. The mean interval between baseline and follow-up was 19 days ±7.2. The mean HSS Pedi-FABS score at follow-up (*n* = 69) was 17.7 ± 5.8. Test-retest reliability of the Dutch HSS Pedi-FABS was sufficient with ICC = 0.76 (*p* < .001). The SEM was calculated at 2.8 points and the SDC at 7.9 points, on a scale from 0 to 30.

### Comparisons between the HSS Pedi-FABS versions

Several differences in psychometric properties were found between the original English, Italian and Dutch versions (Table 5). Compared to the English and Italian version, the Dutch HSS Pedi-FABS showed a lower, but acceptable, test-retest reliability [8, 18]. Compared to the English and Italian HSS Pedi-FABS, the percentage of children scoring the lowest and highest possible scores, indicating floor- or ceiling effect, was lower [8, 18].

**Table 5:** Comparison between the original, Italian and Dutch HSS Pedi-FABS on psychometric properties, adapted from Macchiarola et al. [18]

Psychometric property		Original English HSS Pedi-FABS [8]	Italian HSS Pedi-FABS [18]	Dutch HSS Pedi-FABS	Quality score^a^
Population		10–18 years Athletically active	8–16 years Affected by knee pathologies or deformities	10–18 years Healthy	N/A
Readability		13 years	–	11–12 years	N/A
Content validity		–	–	Target population: 92% of items considered as relevant Professionals: - Construct: < 85% relevance for most items - Target population: > 85% relevance for most items	Positive
Interpretability		Lowest score 0% Highest score 3.9%	Lowest score 19% Highest score 0%	Lowest score 1% Highest score 0%	Positive
Construct validity		Significant positive correlation with ▪ Tegner ▪ Marx ▪ Noyes Sport/Functional ▪ PAQ ▪ Competition level ▪ Current athletic activity (h/week) ▪ Athletic activity during peak season (h/week)	Moderate-to-low correlation with Pedi-IKDC	Significant positive correlation with ▪ PAQ (total) ▪ PAQ-A ▪ Tegner ▪ Competition level	Positive
Reliability	Internal consistency	α = 0.91	α = 0.93	α = 0.82	Positive
	Test-retest reliability	ICC = 0.91	ICC = 0.94	ICC = 0.76	Positive
	SEM	–	SEM 2.1 SDC 5.8	SEM 2.8 SDC 7.9	N/A

^a^ Quality scores for the Dutch HSS Pedi-FABS according to Terwee et al. [21]

*HSS Pedi-FABS* Hospital for Special Surgery Paediatric Functional Activity Scale, *ICC* Intraclass Correlation Coefficient, *N/A* Not Applicable, *PAQ-A/C* Physical Activity Questionnaire – Adolescent/Children, *SDC* Smallest Detectable Change, *SEM* Standard Error of Measurement, “- “stands for not assessed

## Discussion

The most important findings of this study are that the Dutch HSS Pedi-FABS has a good internal consistency, acceptable test-retest reliability, good construct validity and a positive interpretability rating in a Dutch population of healthy paediatric and adolescent participants.

Although the overall interpretation of the psychometric properties was similar, certain differences were found among the HSS Pedi-FABS versions, of which the test-retest reliability and floor and ceiling effects were the most important [8, 18]. These differences may be caused by the differences in interval between T0 and T1, the inclusion criteria and population characteristics. For example, the differences in floor and ceiling effect could be caused by the differences in inclusion criteria [8, 18]. The English HSS Pedi-FABS study included athletically active adolescents, the current study healthy children and adolescents and the Italian study children with knee pathologies [8, 18]. Therefore, it might be expected that the mean score of the English HSS Pedi-FABS study is higher than the Dutch and the Italian, but also that the scores are distributed in the higher score ranges [8, 18]. As the Italian study included children with knee pathologies, the mean score was lower and had a significant floor effect [18]. The mean score of the Dutch HSS Pedi-FABS was more similar to the mean score of the HSS Pedi-FABS in a study on normative data, although a higher floor effect was found in that study [16].

In contrast to the positive ratings on the outcomes of construct validity, interpretability and reliability, content validity showed different results. Content validity among the target population was considered to be good. However, content validity reported by professionals was acceptable for the relevance for the target population, but insufficient for the construct. The questionnaire did not reach 85% relevance on most items for the construct. Besides, 47% of the professionals suggested an additional item to measure physical activity. As multiple issues appeared already in the pre-final version during the pilot study among professionals, several transcultural adaptations were made to solve these issues and the original author was consulted. It was decided to maintain the original form and content of the HSS Pedi-FABS. No additional items were therefore added nor was the content changed.

Compared to other paediatric activity scales, the HSS Pedi-FABS has multiple advantages [8]. The HSS Pedi-FABS is a short and simple scale compared to other questionnaires, that potentially minimizes questionnaire fatigue and increases compliance [8]. Also, the HSS Pedi-FABS is a general measurement of physical activity and not specified on sports or joints, which provides a potential for broader application in clinical outcomes research [8, 16]. In previous studies, the HSS Pedi-FABS has shown to capture changes in physical activity due to recent injury more likely than the Marx Activity Scale, to have more correlations with an athlete’s participation in sports than the Tegner activity scale and to be reliable as patient reported outcome measure (PROM) captured electronically as on paper [33–35].

This study had certain limitations. Criterion validity could not be assessed, as there was no “gold standard” for questionnaires on physical activity [21]. Furthermore, a potential source of bias is the “proxy problem”, as 39% of the children received help from parents in completing the questionnaire [36, 37]. Self-reports of children are not equal to reports by proxy-respondents and a parents’ report can therefore not be substituted for the child’s report [36, 37]. The readability level, however, was estimated to correspond to a readability level of 11- to 12-year-old children [25]. As children and adolescents aged 10 to 18 years were included, it seemed to be a rather high percentage of children receiving help from parents in completing the questionnaire. However, 73% of the children who received assistance were aged between 10 to 12 years and 20% were aged 13 and 14 years. The reasons and type of assistance received was not evaluated. For children who experienced problems in the comprehensibility and who were not able to complete the questionnaire properly, parental assistance might be desirable. This was also advised on the instruction form of the questionnaire. However, whether comprehensibility is the main cause is unknown. It is doubtful whether parental help was necessary for comprehensibility and whether this “proxy problem” might be a source of bias leading to limitations for future use [36, 37]. Besides, most other psychometric properties are good. The current study however, included healthy children and adolescents without a condition or injury limiting their normal physical activity. As the PAMI (Paediatric Anterior cruciate ligament Monitoring Initiative) project focusses on anterior cruciate ligament injuries in children, the current Dutch version is not explicitly validated in that specific population and future research in that specific population is desirable to establish the psychometric properties of the HSS Pedi-FABS. However, previous studies have been conducted in children with knee complaints or pathologies for the English and Italian HSS Pedi-FABS and showed acceptable psychometric properties [18, 33, 35].

## Conclusion

The Dutch HSS Pedi-FABS showed good psychometric properties in a healthy Dutch paediatric and adolescent population. Limitations of the current Dutch HSS Pedi-FABS are content validity on construct of items reported by professionals.

## Supplementary Information

**Additional file 1.** Interviews. Problem codes found in the interviews.

## Data Availability

The datasets used and/or analysed during the current study are available from the corresponding author on reasonable request. The Dutch HSS Pedi-FABS questionnaire is available on request to the authors.

## Abbreviations

ACL: Anterior cruciate ligament
BMI: Body mass index
CCS: Classification coding scheme
FRE: Flesch reading ease
HSS Pedi-FABS: Hospital for Special Surgery Paediatric Functional Activity Brief Scale
ICC: Intraclass correlation coefficient
IOC: International Olympic committee
Máxima MC: Máxima Medical Centre
METC: Medical Ethics Committee
MIC: Minimal Important Change
PAMI: Paediatric Anterior Cruciate Ligament Monitoring Initiative
PAQ-A: Physical Activity Questionnaire for Adolescents
PAQ-C: Physical Activity Questionnaire for Children
PROM: Patient Reported Outcome Measure
SEM: Standard error of measurement
SD: Standard deviation
SDC: Smallest detectable change

## References

[1] Collard DC, Verhagen EA, van Mechelen W, Heymans MW, Chinapaw MJ (2011). Economic burden of physical activity-related injuries in Dutch children aged 10-12. Br J Sports Med.

[2] Jayanthi NA, LaBella CR, Fischer D, Pasulka J, Dugas LR (2015). Sports-specialized intensive training and the risk of injury in young athletes: a clinical case-control study. Am J Sports Med.

[3] Stracciolini A, Casciano R, Levey Friedman H, Meehan WP, Micheli LJ (2013). Pediatric sports injuries: an age comparison of children versus adolescents. Am J Sports Med.

[4] Belechri M, Petridou E, Kedikoglou S, Trichopoulos D (2001). Sports injuries European Union Group. Sports injuries among children in six European union countries. Eur J Epidemiol.

[5] Brophy RH, Lin K, Smith MV (2014). The role of activity level in orthopaedics: an important prognostic and outcome variable. J Am Acad Orthop Surg.

[6] Sylvia LG, Bernstein EE, Hubbard JL, Keating L, Anderson EJ (2014). Practical guide to measuring physical activity. J Acad Nutr Diet.

[7] Janz KF, Lutuchy EM, Wenthe P, Levy SM (2008). Measuring activity in children and adolescents using self-report: PAQ-C and PAQ-A. Med Sci Sports Exerc.

[8] Fabricant PD, Robles A, Downey-Zayas T, Do HT, Marx RG, Widmann RF (2013). Development and validation of a pediatric sports activity rating scale: the Hospital for Special Surgery Pediatric Functional Activity Brief Scale (HSS Pedi-FABS). Am J Sports Med.

[9] Iversen MD, von Heideken J, Farmer E, Rihm J, Heyworth BE, Kocher MS (2016). Validity and comprehensibility of physical activity scales for children with sport injuries. J Pediatr Orthop.

[10] Kocher MS, Smith JT, Iversen MD, Brustowicz K, Ogunwole O, Andersen J (2011). Reliability, validity, and responsiveness of a modified international knee documentation committee subjective knee form (Pedi-IKDC) in children with knee disorders. Am J Sports Med.

[11] Bradley M, Daly A (1994). Use of the logit scaling approach to test for rankorder and fatigue effects in stated preference data. Transportation..

[12] Marx RG, Stump TJ, Jones EC, Wickiewicz TL, Warren RF (2001). Development and evaluation of an activity rating scale for disorders of the knee. Am J Sports Med.

[13] Tegner Y, Lysholm J (1985). Rating systems in the evaluation of knee ligament injuries. Clin Orthop Relat Res.

[14] Hidding LM, Chinapaw MJM, van Poppel MNM, Mokkink LB, Altenburg TM (2018). An updated systematic review of childhood physical activity questionnaires. Sports Med.

[15] Bervoets L, Van Noten C, Van Roosbroeck S, Hansen D, Van Hoorenbeeck K, Verheyen E (2014). Reliability and validity of the Dutch physical activity questionnaires for children (PAQ-C) and adolescents (PAQ-A). Arch Public Health.

[16] Fabricant PD, Suryavanshi JR, Calcei JG, Marx RG, Widmann RF, Green DW (2018). The Hospital for Special Surgery Pediatric Functional Activity Brief Scale (HSS Pedi-FABS): normative data. Am J Sports Med.

[17] Ardern CL, Ekas G, Grindem H, Moksnes H, Anderson A, Chotel F (2018). 2018 International Olympic Committee consensus statement on prevention, diagnosis and management of paediatric anterior cruciate ligament (ACL) injuries. Knee Surg Sports TraumatolArthrosc.

[18] Macchiarola L, Grassi A, Di Paolo S, Pizza N, Trisolino G, Stallone S (2020). The Italian cross-cultural adaptations of the paediatric international knee documentation committee score and the Hospital for Special Surgery Paediatric Functional Activity Brief Scale are reliable instruments in paediatric population. Knee Surg Sports Traumatol Arthrosc.

[19] Moksnes H, Engebretsen L, Seil R (2016). The ESSKA paediatric anterior cruciate ligament monitoring initiative. Knee Surg Sports Traumatol Arthrosc.

[20] Beaton DE, Bombardier C, Guillemin F, Ferraz MB (2000). Guidelines for the process of cross-cultural adaptation of self-report measures. Spine..

[21] Terwee CB, Bot SD, de Boer MR, van der Windt DA, Knol DL, Dekker J (2007). Quality criteria were proposed for measurement properties of health status questionnaires. J Clin Epidemiol.

[22] Zebis MK, Warming S, Pedersen MB, Kraft MH, Magnusson SP, Rathcke M (2019). Outcome measures after ACL injury in pediatric patients: a scoping review. Orthop J Sports Med.

[23] Briggs KK, Lysholm J, Tegner Y, Rodkey WG, Kocher MS, Steadman JR (2009). The reliability, validity, and responsiveness of the Lysholm score and Tegner activity scale for anterior cruciate ligament injuries of the knee: 25 years later. Am J Sports Med.

[24] Eshuis R, Lentjes GW, Tegner Y, Wolterbeek N, Veen MR (2016). Dutch translation and cross-cultural adaptation of the Lysholm score and Tegner activity scale for patients with anterior cruciate ligament injuries. J Orthop Sports Phys Ther.

[25] Douma WH. De Leesbaarheid Van Landbouwbladen. Een Onderzoek Naar en Een Toepassing Van Leesbaarheidsformules. Readability of Dutch Farm Papers. A Summary in English. Afdeling sociologie en sociografie van de landbouwhogeschool Wageningen, Wageningen; 1960.

[26] Mokkink LB, de Vet HCW, Prinsen CAC, Patrick DL, Alonso J, Bouter LM (2018). COSMIN risk of bias checklist for systematic reviews of patient-reported outcome measures. Qual Life Res.

[27] Patrick DL, Burke LB, Gwaltney CJ, Leidy NK, Martin ML, Molsen E (2011). Content validity--establishing and reporting the evidence in newly developed patient-reported outcomes (PRO) instruments for medical product evaluation: ISPOR PRO good research practices task force report: part 2--assessing respondent understanding. Value Health.

[28] Forsyth B, Rothgeb JM, Willis GB, Presser S, Rothgeb JM, Couper MP, Lessler JT, Martin E, Martin J (2004). Does pretesting make a difference? An experimental test. Methods for testing and evaluating survey questionnaires.

[29] McHorney CA, Tarlov AR (1995). Individual-patient monitoring in clinical practice: are available health status surveys adequate?. Qual Life Res.

[30] Ghasemi A, Zahediasl S (2012). Normality tests for statistical analysis: a guide for non-statisticians. Int J Endocrinol Metab.

[31] Tighe J, McManus IC, Dewhurst NG, Chis L, Mucklow J (2010). The standard error of measurement is a more appropriate measure of quality for postgraduate medical assessments than is reliability: an analysis of MRCP (UK) examinations. BMC Med Educ.

[32] van Kampen DA, Willems WJ, van Beers LW, Castelein RM, Scholtes VA, Terwee CB (2013). Determination and comparison of the smallest detectable change (SDC) and the minimal important change (MIC) of four-shoulder patient-reported outcome measures (PROMs). J Orthop Surg Res.

[33] Marom N, Xiang W, Heath M, Boyle C, Fabricant PD, Marx RG (2020). Time interval affects physical activity scores: a comparison of the Marx activity rating scale and the Hospital for Special Surgery Pediatric Functional Activity Brief Scale. Knee Surg Sports Traumatol Arthrosc.

[34] Sabatino MJ, Gans CV, Zynda AJ, Chung JS, Miller SM, Wilson PL (2019). An electronic patient-reported outcomes measurement system in paediatric orthopaedics. J Child Orthop.

[35] Wagner KJ, Sabatino MJ, Zynda AJ, Gans CV, Chung JS, Miller SM (2020). Activity measures in pediatric athletes: a comparison of the Hospital for Special Surgery Pediatric Functional Activity Brief Scale and Tegner Activity Level Scale. Am J Sports Med.

[36] Dietvorst M, Reijman M, van Groningen B, van der Steen MC, Janssen RPA (2019). PROMs in paediatric knee ligament injury: use the Pedi-IKDC and avoid using adult PROMs. Knee Surg Sports Traumatol Arthrosc.

[37] Schmitt LC, Paterno MV, Huang S (2010). Validity and internal consistency of the international knee documentation committee subjective knee evaluation form in children and adolescents. Am J Sports Med.

