# Supplementary material for: Translation and transcultural validation of the Dutch hospital for special surgery paediatric functional activity brief scale (HSS Pedi-FABS)
Source: BMC Musculoskelet Disord. 2021 Oct 6;22:853. doi: 10.1186/s12891-021-04729-0 (PMC8495986; doi:10.1186/s12891-021-04729-0)
Supplement: Supplementary file 1 — Additional file 1. Interviews. Problem codes found in the interviews. [file 12891_2021_4729_MOESM1_ESM.docx]

Supplementary file 1 – Interviews

| Problem codes found in the interviews.  Frequency of codes that were applied to the transcribed interviews | |
| --- | --- |
| **Problem codes** | **Frequency** |
| 1. Comprehension and Communication | 45 |
| Interviewer Difficulties | *9* |
| 1. Inaccurate instruction | 4 |
| 1. Complicated instruction | 2 |
| 1. Difficult to administer | 3 |
| Question Content | *22* |
| 1. Vague topic/term | 10 |
| 1. Complex topic | 9 |
| 1. Topic carried over from earlier question | 0 |
| 1. Undefined term(s) | 3 |
| Question Structure | *14* |
| 1. Transition needed | 0 |
| 1. Unclear respondent instruction | 8 |
| 1. Question too long | 0 |
| 1. Complex, awkward syntax | 0 |
| 1. Erroneous assumption | 6 |
| 1. Several questions | 0 |
| Reference Period | *0* |
| 1. Carried over from earlier question | 0 |
| 1. Undefined | 0 |
| 1. Unanchored or rolling | 0 |
| 1. Memory Retrieval | 8 |
| 1. Shortage of cues | 8 |
| 1. High detail required or information unavailable | 0 |
| 1. Long recall period | 0 |
| 1. Judgment and Evaluation | 10 |
| 1. Complex estimation | 10 |
| 1. Potentially sensitive or desirability bias | 0 |
| 1. Response Selection | 6 |
| Response Terminology | *1* |
| 1. Undefined term(s) | 1 |
| 1. Vague term(s) | 0 |
| Response Units | *0* |
| 1. Responses use wrong units | 0 |
| 1. Unclear what response options are | 0 |
| Response Structure | *5* |
| 1. Overlapping categories | 5 |
| 1. Missing categories | 0 |
| 1. Other | 4 |
| 1. Something else | 4 |
| 1. Relevance | 54 |
| 1. Highly relevant | 37 |
| 1. Somewhat relevant | 13 |
| 1. Not relevant | 1 |
| 1. Unclear | 3 |
| **Total** | **127** |
